# Disassembly Study of Ultrasonically Welded Thermoplastic Composite Joints via Resistance Heating

**DOI:** 10.3390/ma14102521

**Published:** 2021-05-12

**Authors:** Harry Frederick, Wencai Li, Genevieve Palardy

**Affiliations:** Department of Mechanical and Industrial Engineering, Louisiana State University, 3261 Patrick F. Taylor Hall, Baton Rouge, LA 70803, USA; hfrede6@lsu.edu (H.F.); wli45@lsu.edu (W.L.)

**Keywords:** thermoplastic composites, ultrasonic joints, resistance heating

## Abstract

This manuscript explores the disassembly potential of ultrasonically welded thermoplastic composite joints for reuse or recycling through resistance heating via a nanocomposite film located at the welded interface. Nanocomposite films containing multi-walled carbon nanotubes (MWCNTs) were characterized for thermo-electrical behavior to assess self-heating. It was generally observed that maximum temperature increased with MWCNT and film thickness. To demonstrate potential for disassembly, glass fiber/polypropylene adherends were welded with nanocomposite films. Shear stress during disassembly was measured for three initial adherend’s surface temperatures. It was found that the required tensile load decreased by over 90% at the highest temperatures, effectively demonstrating the potential for disassembly via electrically conductive films. Fracture surfaces suggested that disassembly was facilitated through a combination of nanocomposite and matrix melting and weakened fiber–matrix interface. Limitations, such as slow heating rates and the loss of contact at the interface, imply that the method could be more suited for recycling, instead of repair and reuse, as the heat-affected zone extended through the adherends’ thickness at the overlap during heating.

## 1. Introduction

Thermoplastic composites (TPCs) are used in several industries, such as automotive, aerospace, and wind energy, because of their high specific modulus and strength, fracture toughness, damage tolerance, and impact and corrosion resistance [1,2,3,4]. Common thermoplastic matrices include polypropylene (PP), polyethylene (PE), and polycarbonate (PC), used in a range of low-cost applications. High-performance thermoplastic matrices encompass higher temperature polymers, such as polyamide 6 (PA6), polyetherimide (PEI), polyphenylene sulfide (PPS), and polyether ether ketone (PEEK) [5]. In addition, Arkema recently developed a liquid thermoplastic resin with similar mechanical properties to thermosetting resins, Elium^®^, which enables the use of composite manufacturing technologies traditionally used for thermosets [6]. As thermoplastics can be thermoformed when heated up above certain temperatures, TPCs have the potential for recycling, reuse and reshaping into new components, as well as joining through fusion bonding [7]. The latter can eliminate the use of rivets, reducing weight, cost and stress concentration. It is more time-efficient than thermoset adhesive bonding because it does not require surface preparation. Fusion bonding is categorized into thermal, electromagnetic and friction welding [3]. Ultrasonic welding (USW) is a technique that has gained momentum in the past few years for TPCs, as it is fast, energy-efficient, and suitable for spot and continuous joining configurations [8,9,10,11].

USW joins adherends by the application of high frequency, low amplitude vibrations through a sonotrode (or horn) to generate heat via frictional and viscoelastic mechanisms [12,13]. An “energy director” (ED) must usually be placed at the weld interface to concentrate heat generation. Triangular protrusions are typically employed in the plastics industry, but for continuous fiber-reinforced thermoplastics, thin films are also suitable and lead to high strength welds [14,15,16,17,18]. Many studies in the literature experimentally investigated the effect of process parameters (amplitude, force and control mode) and ED geometry on bond quality [10,14,15,19,20,21,22,23,24,25,26] and heat generation [18,27,28]. For instance, it was reported that using the vertical displacement of the sonotrode could lead to consistent weld quality using power and displacement curves from the welder. On the other hand, energy director-less welding was found to be possible when controlling the process through time. The prediction of temperature profiles, consumed power, ED flow, and bond strength has shown reasonable accuracy through multi-physics modeling and artificial intelligence methods [13,29,30,31,32,33,34,35].

While the USW process and bond strength have been extensively studied for a wide range of TPCs, there is limited research on structural health monitoring and repair of joints. Prior research has shown the potential for multifunctional, nanocomposite films as EDs for USW [36,37]. Those films, rendered electrically conductive by the addition of multi-walled carbon nanotubes (MWCNTs), enabled USW and structural health monitoring at the welded interface through electrical resistance changes. Another function they could fulfill is localized resistance heating at the interface to facilitate disassembly and repair.

Nanocomposite-based heating elements were recently developed and successfully used for the resistance welding of TPCs, notably by Brassard et al. [38,39]. MWCNT/PEI nanocomposite films with weight fractions up to 15 wt.% led to an electrical conductivity of 0.92 S/cm. With 10 wt.% MWCNT, the films reached the glass transition temperature (>217 °C) at an applied voltage of 25 V, demonstrating their Joule heating behavior. However, infrared camera monitoring revealed non-uniform temperature distribution, likely due to copper electrodes acting as heat sinks. In the literature, a wider range of studies on nanocomposite films as susceptors for the induction welding of TPCs or induction heating of adhesives have been carried out. Farahani et al. showed that silver nanoparticle-based thermoplastic films are suitable as susceptors for induction welding, reaching melting temperature in less than 50 s at 400 A [40,41]. However, the potential for disassembly and the repair of fusion bonded TPC joints has not been investigated in the literature.

Although they have not been used to join TPCs, reversible adhesives were developed to facilitate disassembly and the healing of thermoset composite adherends [42,43]. Those adhesives are made of a thermoplastic matrix, containing ferromagnetic nanoparticles to induce temperature increase through induction heating. Reversible joints provide the benefits of both adhesive and mechanically fastened techniques, including ease of disassembly. The method was demonstrated with acrylonitrile butadiene styrene (ABS) and up to 20 wt.% ferromagnetic nanoparticles, but is so far limited to fiber-reinforced epoxy composites. This means the adherends would not be significantly affected during the process. In the fusion bonding of TPCs, the disassembly procedure would be expected to affect the adherends, as the bond line is made of the same thermoplastic as the adherends. To the best of the authors’ knowledge, disassembly studies on welded TPC joints have not been reported in the literature. Therefore, the aim of this research work is to address this gap by focusing on two particular topics: (1) assess ease of disassembly for ultrasonically welded joints by investigating effect of resistance heating temperature; (2) understand disassembly mechanisms and the extent to which adherends are affected during the process.

This study will demonstrate the potential for disassembly of ultrasonically welded TPC joints via resistance heating. First, the thermo-electrical characterization of MWCNT-based nanocomposite films containing different filler weight fractions was investigated to assess their use as heating elements. Second, ultrasonic welding was used to assemble glass fiber/polypropylene adherends into a single lap joint configuration. Disassembly was carried out with a tensile testing apparatus under a range of applied voltages at the interface, leading to different interface temperatures. Third, the behavior of the joints during the disassembly procedure was analyzed through shear stress and temperature curves, fractography analysis and extent of heat-affected zone. Finally, the limitations of this technique and proposed future research directions will be discussed.

## 2. Materials and Experimental Methods

### 2.1. Materials

Polypropylene (PP) masterbatches containing multi-walled carbon nanotubes (MWCNTs) were used for this study. MWCNT loading ratios equal to 15 wt.%, 20 wt.%, and 25 wt.% were purchased from Cheap Tubes Inc (Brattleboro, VT, USA). Those specific ratios were selected based on preliminary Joule heating experiments, previous work demonstrating suitability for ultrasonic welding [36,37] and literature on high content CNT-based polymer films for sensing, self-heating and resistance welding [38,39,44,45].

Glass fiber/polypropylene (GF/PP) adherends were used for ultrasonic welding and disassembly testing. GF/PP IE 6030 unitape Polystrand^TM^ prepregs with a fiber volume fraction of 60%, an areal weight of 461 g/m^2^ and a tape thickness of 0.33 mm were purchased from Avient (formerly PolyOne, Englewood, CO, USA).

### 2.2. Nanocomposite Films and Thermoplastic Composites Fabrication

The nanocomposite films (MWCNT/PP) were manufactured with a heated laboratory press (Dake, Grand Haven, MI, USA). During compression molding, PTFE (polytetrafluoroethylene)-coated fiberglass release films and steel shims were placed between the heated platens to produce a consistent surface finish and to control the films’ final thickness. Based on previous work on the effect of ED thickness on USW of TPCs [10,16,46], shims with thicknesses equal to 0.06 mm, 0.25 mm and 0.50 mm were selected. The molded nanocomposite films were cut into rectangular specimens and stored in sealed bags. For thermo-electrical characterization, the films dimensions were 50 mm × 15 mm, while they were 30 mm × 25 mm for ultrasonic welding.

GF/PP substrates were manufactured by compression molding with a laboratory press (Dake, Grand Haven, MI, USA). Eight unidirectional prepreg layers, measuring 254 mm × 254 mm, were stacked in a [0]_8_ sequence between steel plates, then placed between the press’ heated platens. The laminate was consolidated under 1 MPa at 180 °C for approximately 15 min. During compression molding, a thermocouple was placed at one edge of the laminate to monitor the temperature between the plies. After demolding, a laminate with a final thickness between 1.8 mm and 1.9 mm was obtained. Prior to welding, the laminate was cut into rectangular specimens (101.6 mm × 25.4 mm) with a water-cooled diamond saw (PICO 155 from Pace Technologies). The longer side was cut along the direction of the glass fibers.

### 2.3. Thermo-Electrical Characterization

**Figure 1:**
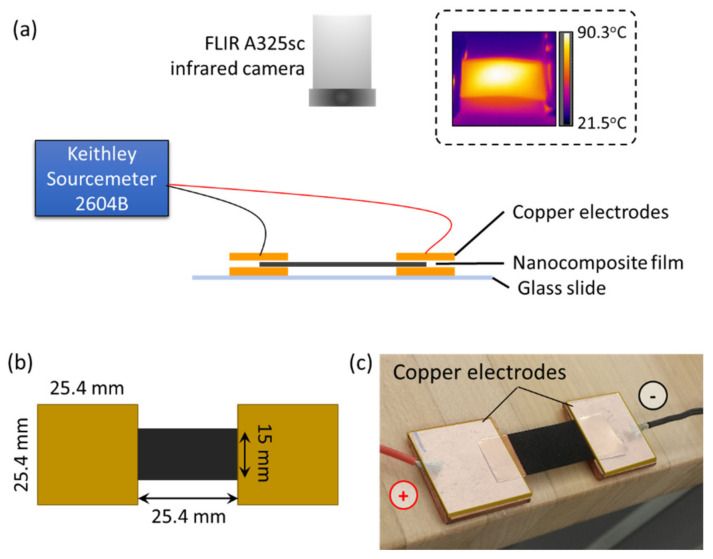
(**a**) Overall setup used for electrical conductivity and Joule heating measurements. Inset shows representative 2D temperature plot recorded with infrared camera at 10 V for a 0.50 mm-thick 20 wt.% MWCNT/PP film after approximately 1 min; (**b**) copper electrodes and nanocomposite film dimensions; (**c**) example of actual nanocomposite film and electrodes placement. Dimensions are not to scale.

Electrical conductivity and the resistance heating measurement setup is illustrated in Figure 1a. A voltage was applied through copper electrodes placed at both ends of the films with a Keithley Sourcemeter 2604B (maximum voltage and current of 40 V and 1 A, respectively), shown in Figure 1b,c. The DC voltages used to gather electrical and thermal data were 1 V, 2 V, 4 V, 6 V, 8 V, and 10 V. All film thicknesses were tested (0.06 mm, 0.25 mm, and 0.50 mm) to assess their effect on thermo-electrical behavior. The electrical conductivity of the films (*σ*, in S/cm) was calculated using Equation (1), shown below, where *R_Avg_* is the average resistance (in Ohms), *L* is the length of the film between electrodes (in cm), and *A* is the cross-sectional area of the film (in cm^2^):

(1) $$\sigma =\frac{L}{R_{Avg}A}$$

For resistance heating, each voltage was applied for three minutes with 30 s between voltages, while the Keithley KickStart software (version 2.0, Beaverton, OR, USA) acquired resistance, power and current data at a rate of 10 data points/second. Two-dimensional temperature plots were acquired with a FLIR A325sc infrared camera (FLIR Systems, Spicewood, TX, USA) placed above the film, at a rate of 15 Hz (example shown in Figure 1a in inset). Temperature profiles were extracted at the center of the film. For each applied voltage, MWCNT weight fraction and film thickness, seven to ten samples were tested.

### 2.4. Ultrasonic Welding Procedure

**Figure 2:**
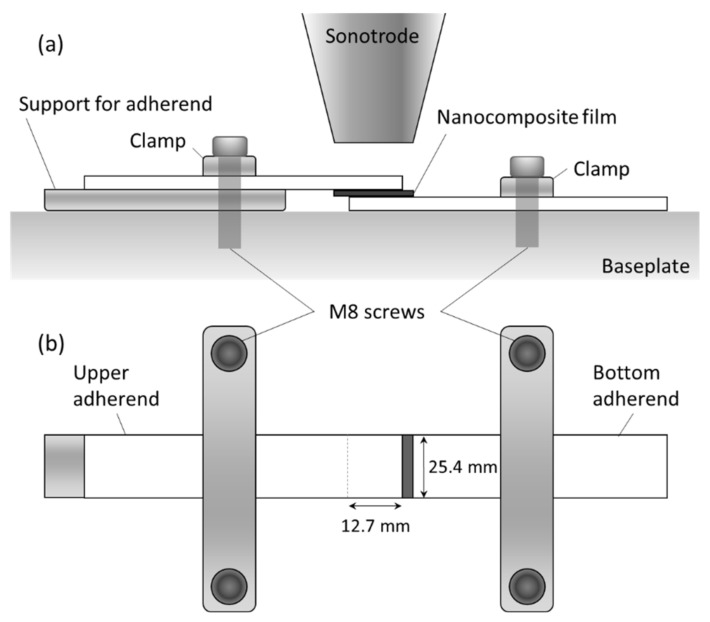
Schematic of ultrasonic welding fixture used in this study for GF/PP adherends and MWCNT/PP energy director films: (**a**) side view and (**b**) top view. Dimensions are not to scale.

GF/PP adherends were welded in a single lap configuration with an overlap area of 25.4 mm × 12.7 mm. A Dynamic 3000 ultrasonic welder (Rinco Ultrasonics, Danbury, CT, USA) with a maximum power of 3000 W and a constant operating frequency of 20 kHz was used with a 40 mm diameter titanium sonotrode. The booster and sonotrode gains were 1:1.5 and 1:3.85, respectively. Both adherends were clamped with aluminum bars and M8 socket head screws on a baseplate, as shown in Figure 2a,b. A nanocomposite film was placed between the adherends to act as the energy director. Films containing 15 wt.%, 20 wt.%, and 25 wt.% MWCNT with a thickness of 0.50 mm were used. Even though thermo-electrical characterization was performed on three different thicknesses, the thickest films were selected for welding and disassembly because they led to the highest bond line thickness, as will be discussed in Section 4.1.1.

For all welds, during the vibration phase, a force of 1000 N and an amplitude of 38.1 μm were applied. The duration of the vibration phase was controlled through the vertical displacement of the sonotrode (also called “travel”) until it reached a value equal to 60% of the initial films’ thickness. After reaching the prescribed travel value, a consolidation force of 1000 N, for a duration of 4 s, was applied. Those welding parameters were chosen based on previous research [36,37]. The power and travel curves with respect to welding time were acquired for each weld.

### 2.5. Disassembly of Welded Joints

Following ultrasonic welding, excess polymer at the interface edges was removed with a razor blade. Two 30 AWG copper wires were connected at the interface with silver paint (SPI #05002-AB, electrical resistivity of 1.2 × 10^−4^ Ohms·cm) to maximize electrical contact. Painted wires and interface were dried overnight before disassembly experiments.

**Figure 3:**
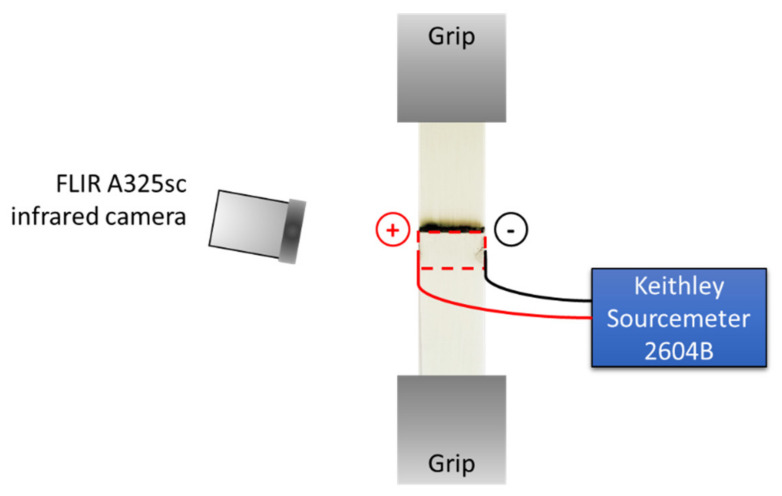
Disassembly setup with infrared camera and sourcemeter. The dashed rectangle indicates the surface area that was monitored for temperature during the tests. Not to scale.

In order to quantify the shear stress required to disassemble the welded joints, the samples were tested with a 50 kN tensile machine (TestResources 313, TestResources Inc., Shakopee, MN, USA), according to ASTM D1002. A schematic of the test setup is shown in Figure 3. The specimens were clamped between hydraulic grips at a distance of 60 mm. The position of both grips was adjusted so that the load direction was aligned with the overlap direction. A voltage between 14 V and 20 V was applied while monitoring the surface temperature of the GF/PP adherend with a FLIR A325sc infrared camera (FLIR Systems, Spicewood, TX, USA). While an external monitoring method does not provide the same accuracy as an embedded sensor at the interface, it was selected for two reasons: (1) the ultrasonic welding process may affect the position and integrity of embedded sensors at the interface due to ultrasonic vibrations (e.g., thermocouples or fiber optic sensors); (2) temperature measurements might become inaccurate as failure initiation and propagation occurs at the interface. Disassembly was initiated at a loading rate of 1.3 mm/min when the surface temperature reached either 110 °C, 130 °C, or 150 °C at the center point of the 25.4 mm × 12.7 mm overlap (delineated by a red, dashed rectangle in Figure 3). Those three temperature values were selected based on simplified 3D thermal analyses, detailed in Section 3 of this manuscript. After disassembly, the extent of the heat-affected zone (HAZ) was quantified using image analysis with the ImageJ software (National Institutes of Health, NIH, version 1.53e, Bethesda, MD, USA). The HAZ area was measured and its % value was calculated with respect to the overlap area.

### 2.6. Scanning Electron Microscopy (SEM)

After disassembly, the microstructure of the fracture surfaces was observed through SEM. Surfaces were coated with gold using a sputter coater (EMS550X, Electron Microscopy Sciences, Hatfield, PA, USA) under a vacuum of 10^−1^ mbar, at 25 mA for 2 min. A high-performance JSM-6610LV SEM (JEOL Ltd., Tokyo, Japan) was employed to capture images, at an acceleration voltage of 15 kV.

## 3. Prediction of Heat-Affected Zone for Disassembly Experiments

Despite the low thickness of the bond line (≤0.50 mm) and adherends (<1.90 mm), a lag was expected between the surface temperature and the actual temperature at the welded interface. A simplified 3D thermal analysis was carried out in the SolidWorks Thermal Simulation module to predict the temperature at the surface of the adherend (as shown in Figure 3) for various interface temperatures. The goal of these analyses was to provide a range of surface temperatures that would guide the design of the disassembly experiments, based on material properties from the suppliers and found in the literature.

**Figure 4:**
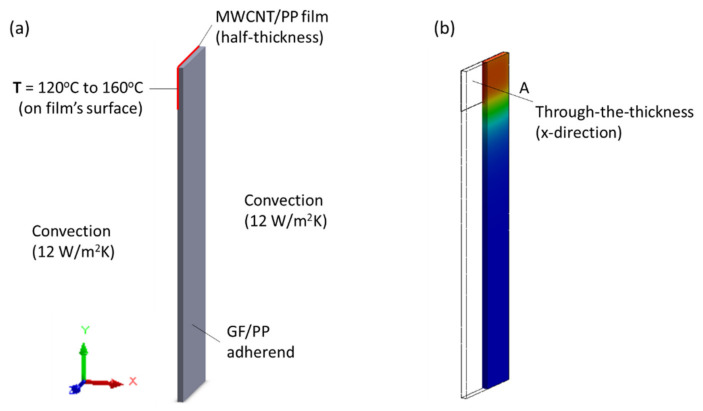
(**a**) Boundary conditions for finite element analysis used for prediction of temperature profile through the thickness of GF/PP joint. Symmetry was assumed along the ZY plane; and (**b**) Example of 3D thermal plot with location of plotted results at the mid-plane, along x-direction at center of bond line.

Figure 4a shows the boundary conditions for the thermal finite element analysis (FEA). The thickness of the bond line was 0.1 mm (half-thickness of MWCNT/PP interface) and the GF/PP adherend was 1.8 mm thick. A forced air convection coefficient of 12 W/m^2^·K was selected for the room in which tests were to be carried out. It was applied to all adherend surfaces in contact with air. The contact between ED and adherend was defined as “Bonded”. The temperature at the interface was set at three values: 120 °C, 140 °C or 160 °C, based on the melting temperature of GF/PP adherends and MWCNT/PP films, between 140 °C and 150 °C [36]. Due to the orthotropic behavior of the adherends (UD layup), two thermal conductivity values (*k_y_*, *k_x_* = *k_z_*) were estimated using the rule of mixture shown in Equation (2) and Equation (3):

(2) $$k_{y}\;=\;\left(1-V_{GF}\right)k_{PP}+V_{GF}k_{GF},$$

(3) $$\frac{1}{k_{x,z}}=\frac{\left(1-V_{GF}\right)}{k_{PP}}+\frac{V_{GF}}{k_{GF}}$$

where *V_GF_* is the glass fiber volume fraction, *k_PP_* is the thermal conductivity of polypropylene (in W/m·K) and *k_GF_* is the thermal conductivity of glass fibers (in W/m·K). The material properties for the GF/PP adherends are listed in Table 1. The heat capacity, *C_p_* (in J/kg·K), was calculated based on the rule of mixture, as described in Equation (2). The MWCNT/PP films were assumed to exhibit isotropic properties with random carbon nanotubes orientation and distribution. The main thermal properties are listed in Table 1 with the corresponding references in the literature. The nanocomposite films’ thermal conductivity was estimated to range between 0.55 W/m·K and 0.65 W/m·K based on [47,48], to account for MWCNT weight fraction and potential variations in dispersion.

**Figure 5:**
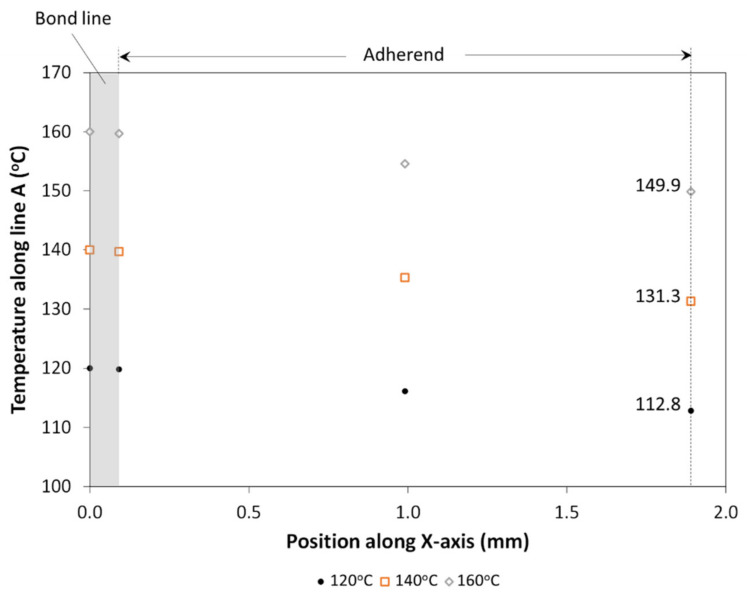
Predicted through-the-thickness temperature profiles along line A at the cross-section labeled in Figure 4b when interface is set at a temperature of 120 °C, 140 °C and 160 °C. The bond line material was 15 wt.% MWCNT/PP.

Figure 5 shows the through-the-thickness temperature profiles along line A at the cross-section labeled in Figure 4b. The results for 15 wt.% MWCNT/PP film at the bond line are presented, but no significant differences were found for the range of *k_CNT/PP_* values in Table 1. The surface temperature at the center point of the overlap is 112.8 °C, 131.3 °C and 149.9 °C for an interface temperature of 120 °C, 140 °C and 160 °C, respectively. Given a temperature gradient around 10 °C, as well as the assumptions and simplifications made for thermal analysis, it was estimated that the disassembly experiments should be carried out when the surface temperature of the GF/PP adherend reached 110 °C, 130 °C and 150 °C to adequately capture the behavior of the heated joint.

## 4. Experimental Results and Discussion

### 4.1. Nanocomposite Films Characterization

#### 4.1.1. Electrical Conductivity

**Figure 6:**
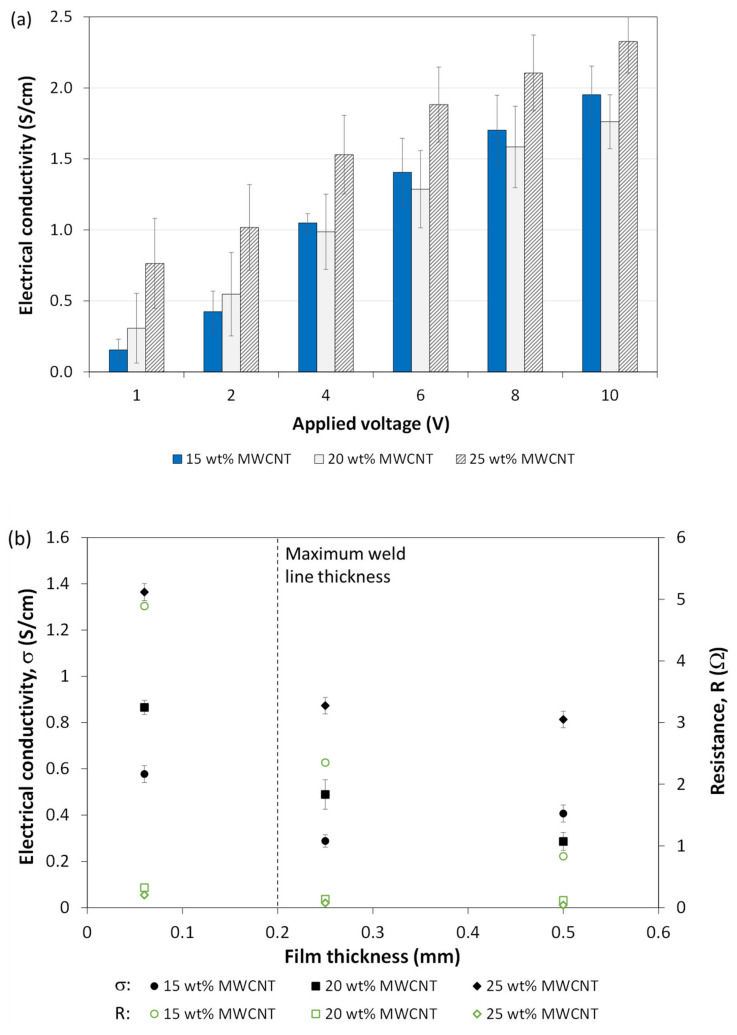
(**a**) Influence of applied voltage on average film electrical conductivity for 15 wt.%, 20 wt.% and 25 wt.% MWCNT; (**b**) influence of film thickness on electrical conductivity (filled markers) and resistance (unfilled markers) for 15 wt.%, 20 wt.% and 25 wt.% MWCNT. Representative data shown when voltage of 2 V was applied.

Figure 6a shows results for electrical conductivity measurements of nanocomposite PP films containing 15 wt.%, 20 wt.%, and 25 wt.% MWCNT, across all three thicknesses. Overall, values are in the same order of magnitude as previously observed in the literature for MWCNT/PP films [49,50,51], as well as for nanocomposite heating elements designed for resistance welding with weight fractions above 10 wt.% MWCNT [39]. Two general trends are observed. First, for the same applied voltage, average conductivity generally increased with CNT weight fraction, more prominently at 1 V and 2 V. Second, for the same weight fraction, conductivity increased with applied voltage, which indicates non-ohmic behavior. This is consistent with the literature, where it was observed that MWCNT nanocomposites exhibit tunneling conductive mechanisms, where a stronger applied electric field creates more conductive pathways through the material [52].

However, due to the large standard deviation caused by the variation in resistance measurements during this time period, statistical significance between means at different voltages and weight fractions was assessed using a two-way analysis of variance (ANOVA), followed by Tukey’s multiple comparison test. The software GraphPad Prism 9.1.0 was used to carry out statistical analyses. The level of significance was set at *p* < 0.05. At 1 V and 2 V, the only significant comparisons (*p* < 0.05) were between 15 wt.% and 25 wt.% MWCNT. At all other voltages, 20 wt.% versus 25 wt.% MWCNT was significant. For the same wt.% value, the main ANOVA outcomes can be summarized as follows: no significance was determined between 1 V and 2 V, then between 6 V and 8 V, 6 V and 10 V, and 8 V and 10 V. Other comparisons between 1 V vs. 4 V, 6 V, 8 V and 10 V, between 2 V vs. 6 V, 8 V and 10 V, then between 4 V vs. 8 V and 10 V, were determined to be significant. Thus, this confirms the general increasing trend with MWCNT weight fraction and applied voltage.

The effect of film thickness was assessed separately and a representative plot is shown in Figure 6b at an applied voltage of 2 V. Thickness is especially important with respect to the welding process as it is controlled through the vertical displacement (travel). In our study, a travel equal to 60% of the initial film thickness (0.50 mm) was used, meaning the final bond line thickness would be equal to 0.20 mm at most. For all MWCNT fractions, the general trend shows a decrease in conductivity with an increase in film thickness. This is opposite to what was observed in the literature for CNT/PDMS nanocomposites prepared with a centrifugal mixer [53]. However, the fabrication method used in our study, compression molding, can explain this behavior. As no additional mechanical mixing or solvent-based dissolution was employed, the CNT dispersion is likely not perfectly random and uniform across the thickness. This may affect the density of the conducting channels in the CNT network and in turn, the electrical conductivity.

Corresponding resistance values are reported in Figure 6b as well. As will be explained in Section 4.1.2, lower resistance leads to higher temperature increase through Joule heating. This indicates that higher MWCNT fractions or thicknesses would allow for reaching higher temperatures for the same applied voltage. Based on the trend shown in Figure 6b, a higher thickness at the weld line would be preferable for the disassembly procedure to insure the desired temperatures can be reached. Therefore, as was described in Section 2.4., the thickest nanocomposite films (0.50 mm) were used as energy directors for the ultrasonic welding process, leading to the highest bond line thickness.

#### 4.1.2. Resistance Heating

Resistance heating follows Joule’s Law, as shown by Equation (4), where *P* represents Watts of heating, *I* is the applied current (in A), *V* is the applied voltage (in V), and *R* is the electrical resistance (in Ohms) [54].

(4) $$P=IV=\frac{V^{2}}{R}\;,$$

**Figure 7:**
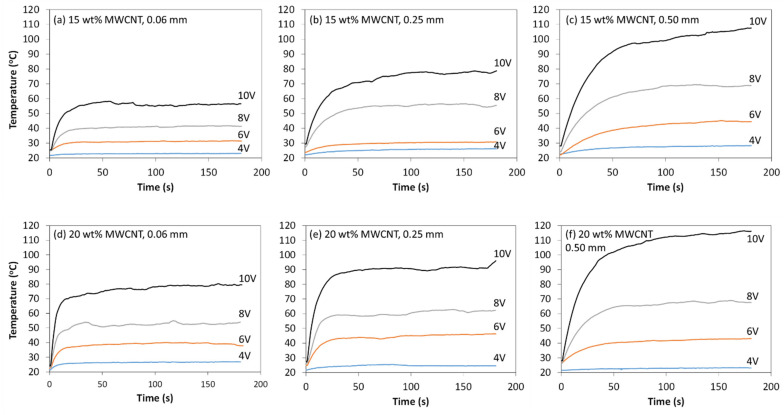
Temperature profiles of MWCNT/PP nanocomposite films at different input voltages and thicknesses: (**a**–**c**) 15 wt.% MWCNT, 0.06 mm, 0.25 mm and 0.50 mm thicknesses, respectively; and (**d**–**f**) 20 wt.% MWCNT, 0.06 mm, 0.25 mm and 0.50 mm thicknesses, respectively.

**Figure 8:**
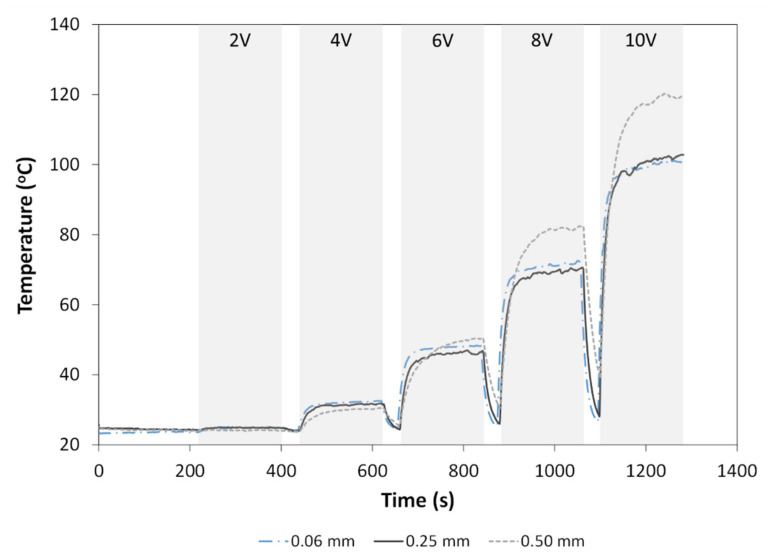
Representative temperature profiles measured for 25 wt.% MWCNT/PP films when voltages from 2 V to 10 V are applied for three minutes each.

As Equation (4) shows, for any given voltage the amount of heating is controlled by the resistance of an object. Therefore, the lower the resistance, the more heat it will generate. Moreover, under a given voltage, thicker films would heat up more because, as shown by Equation (5) below, the resistance of an object will decrease with a larger cross-sectional area:

(5) $$R=\rho \frac{L}{A}\;,$$

where *ρ* is the resistivity, *L* is the length (in mm), and *A* is the cross-sectional area (in mm^2^). Examples of thermal profiles measured for nanocomposite films containing 15 wt.% and 20 wt.% MWCNT are shown in Figure 7. The maximum temperature generally increased with film thickness, CNT weight fraction and applied voltage (Equation (4)). In some cases, slight deviations from this trend were expected based on the large standard deviations seen in Figure 6a and resulting resistance values. At 15 wt.% MWCNT, maximum temperatures of 58.0 °C, 78.7 °C and 108.0 °C were obtained at 10 V, for 0.06 mm, 0.25 mm and 0.50 mm thickness, respectively. On the other hand, at 20 wt.% MWCNT, maximum temperatures of 80.2 °C, 96.1 °C and 116.0 °C were obtained at 10 V, for 0.06 mm, 0.25 mm and 0.50 mm thicknesses, respectively. Figure 8 shows a composite of the measured temperature profiles during the entire Joule heating experiment, when voltage was increased from 2 V up to 10 V for all film thicknesses. Maximum temperatures were obtained at 10 V with 101.6 °C, 102.8 °C and 120.2 °C for 0.06 mm, 0.25 mm and 0.50 mm film thicknesses, respectively.

For the GF/PP adherends and MWCNT/PP films used in this study, their melting temperature (*T_m_*) was measured by differential scanning calorimetry (DSC) in a previous study [36]. The adherends’ *T_m_* was 150 °C, while the *T_m_* of the MWCNT/PP films varied between 141 °C and 149 °C. For the purpose of disassembly, it is expected an interface temperature close to, or slightly above, this range of temperature should be reached through the energy director (MWCNT/PP film). As previously mentioned in Section 4.1.1, the bond line thickness is expected to be equal to 0.2 mm at most. Therefore, an applied voltage above 10V would be required for disassembly experiments, based on the trends observed in Figure 7 and Figure 8.

### 4.2. Disassembly Study of Ultrasonically Welded Joints

#### 4.2.1. Tensile Test Results 

**Figure 9:**
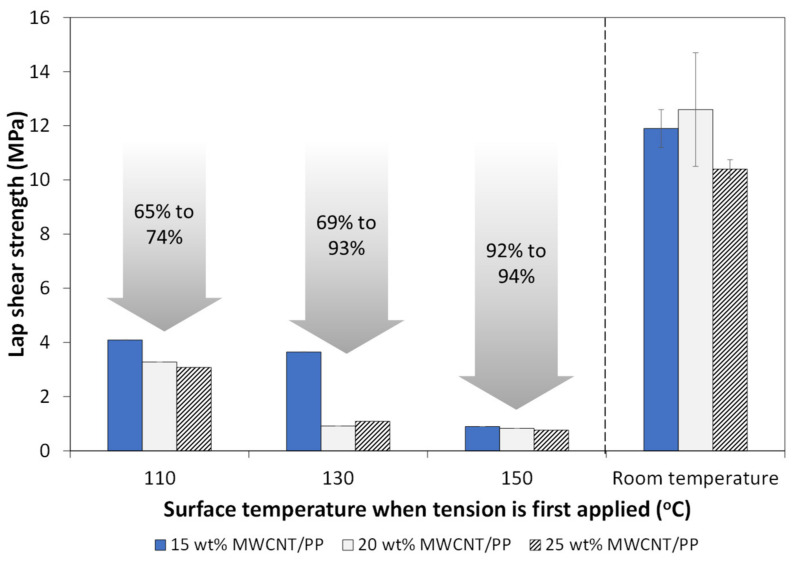
Comparison between lap shear strength of GF/PP welded joints during disassembly procedure when surface temperature reached 110 °C, 130 °C and 150 °C. Interface contained 15 wt.%, 20 wt.% and 25 wt.% MWCNT. Room temperature values are used as a reference, as reported in [36].

Feasibility of the disassembly procedure was assessed and quantified using a tensile testing machine. The lap shear strength (LSS) from load–displacement curves was calculated using the maximum load and the overlap area (25.4 mm × 127 mm). For each MWCNT weight fraction, disassembly was initiated at three adherend’s surface temperatures: 110 °C, 130 °C and 150 °C. Figure 9 summarizes the calculated LSS for all cases and the range of LSS reduction when compared to room temperature tests. It is observed that, as surface temperature increased to 150 °C, the required strength for disassembly was reduced by up to 94%, corresponding to an applied tensile load below 250 N. The lowest surface temperature (110 °C) led to a considerable reduction in LSS, but as the interface temperature likely did not reach melting point, it is not as effective as higher temperatures. At 130 °C, there is a considerable difference between 15 wt.% MWCNT and 20 wt.% or 25 wt.% MWCNT films. There are potentially two causes for this behavior: (1) it was observed that an increase in MWCNT content could lead to lower toughness at the interface for welded joints [36,39,40]; (2) due to the slow crosshead speed during disassembly tests (1.3 mm/min), the temperature likely continued to increase at the interface, which may have been more significant at higher MWCNT loadings.

**Figure 10:**
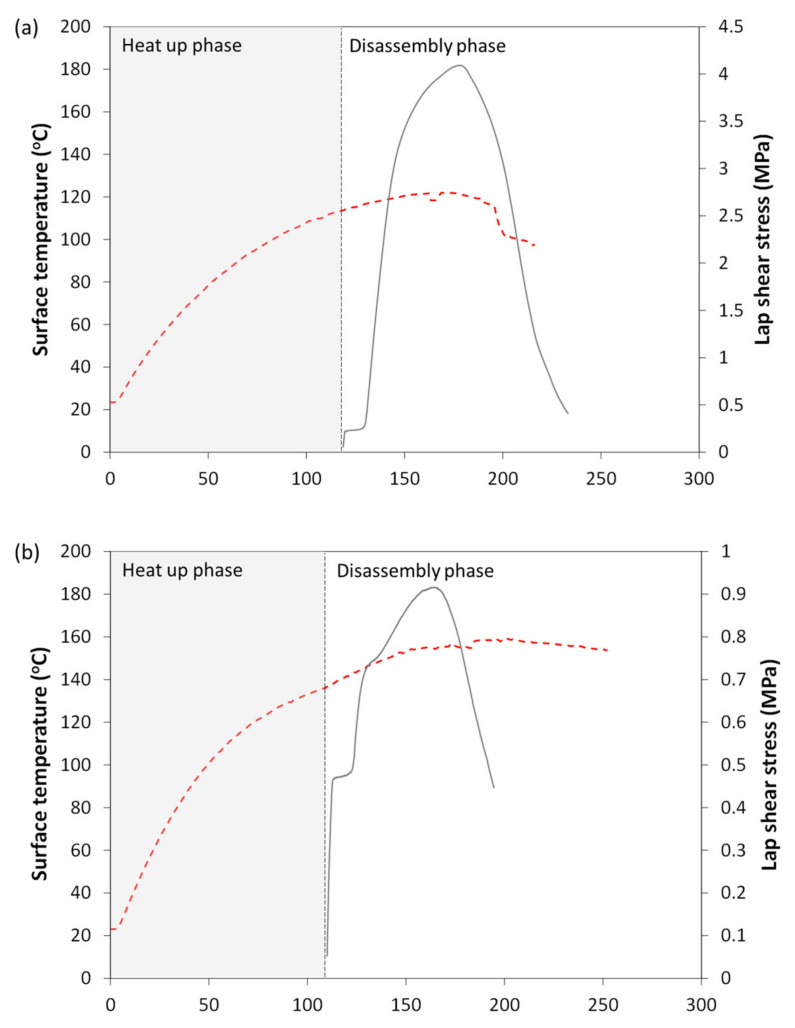

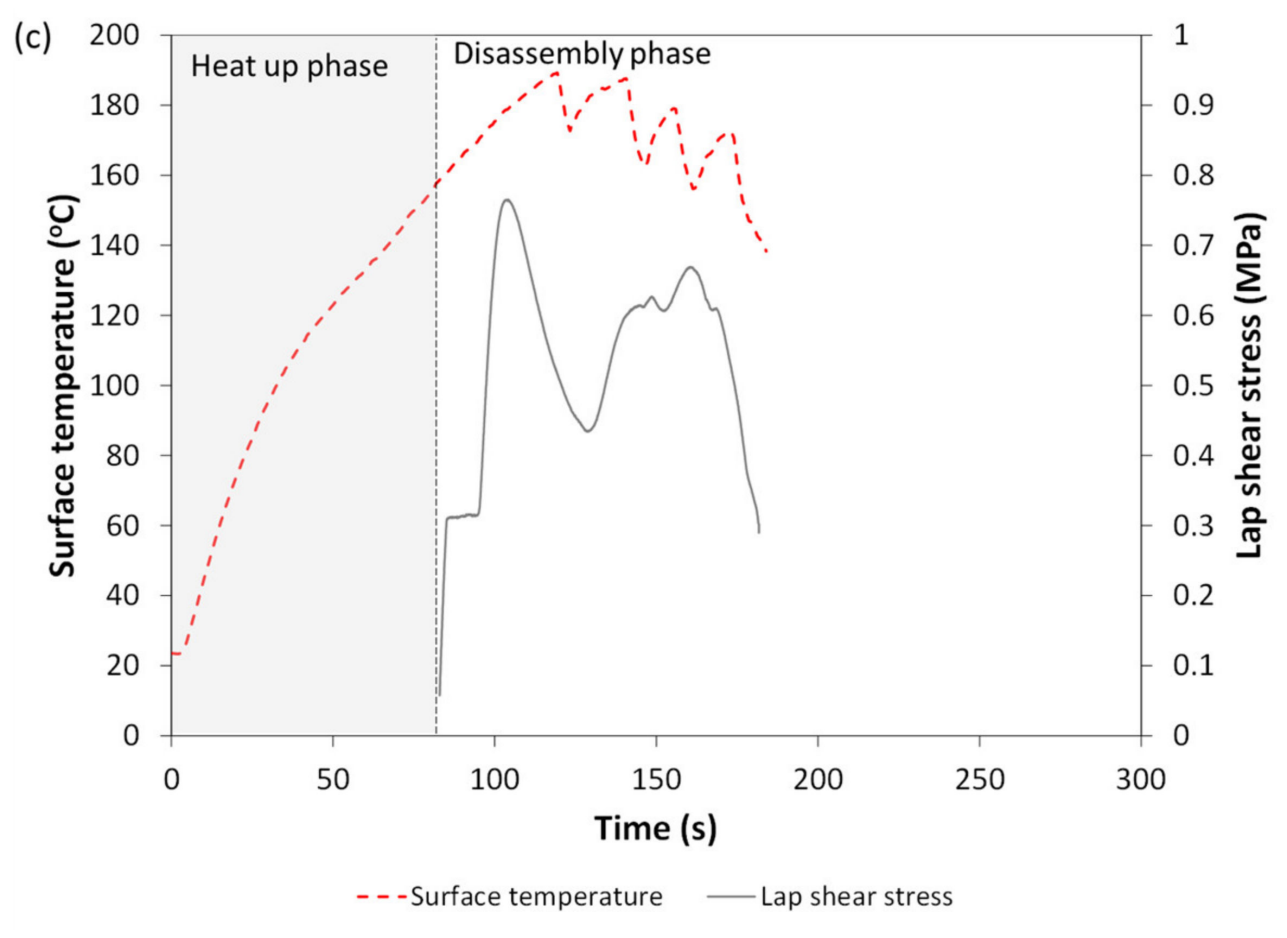
Representative lap shear stress curves (solid lines) of GF/PP welded joints during disassembly procedure with interface containing (**a**) 15 wt.% MWCNT at adherend’s surface temperatures of 110 °C; (**b**) 20 wt.% MWCNT at adherend’s surface temperatures of 130 °C; and (**c**) 25 wt.% MWCNT at adherend’s surface temperatures of 150 °C. Corresponding surface temperature curves are shown as dashed lines.

To further investigate the joints’ behavior during disassembly, the shear stress and surface temperature curves were simultaneously plotted with respect to time, as seen in Figure 10. Representative curves are shown for all weight fractions on Figure 10a–c (15 wt.%, 20 wt.% and 25 wt.% MWCNT), at one surface temperature (110 °C, 130 °C and 150 °C). The heat up and disassembly phases are labeled to show the duration of each one. All tests were initiated after less than two minutes (120 s), with the fastest heat up phase for 25 wt.% MWCNT/PP films. Similarly, the disassembly phase generally lasted less than two minutes. As the applied load increased at the beginning of the disassembly phase, the surface temperature, and by extension, interface temperature, continued to increase as well because the contact at the weld line was not yet severed. However, after failure initiation (at the stress peak), the temperature slowly started to decrease as the integrity of the interface was compromised, leading to fewer conductive paths between MWCNTs. Since the applied voltage was kept constant throughout the disassembly procedure, the temperature consequently decreased. In some cases, as seen in Figure 10c, the disassembly phase displayed an inconsistent stress curve. One possible explanation is that, upon closer inspection of the specimens after disassembly, a small crack defect along the direction of the fibers was found in the adherends at the overlap. As all adherends were visually inspected after welding and no such defect was detected, it is reasonable to assume the crack was created during the disassembly process, likely explaining the inconsistent curves seen in Figure 10c. Another explanation is the reduced heating capacity resulting from failure initiation at the interface, and leading to cool down, as observed in the temperature curve. Cool down could contribute to an increase in stress during the process.

#### 4.2.2. Fractographic and Heat-Affected Zone Analysis

**Figure 11:**
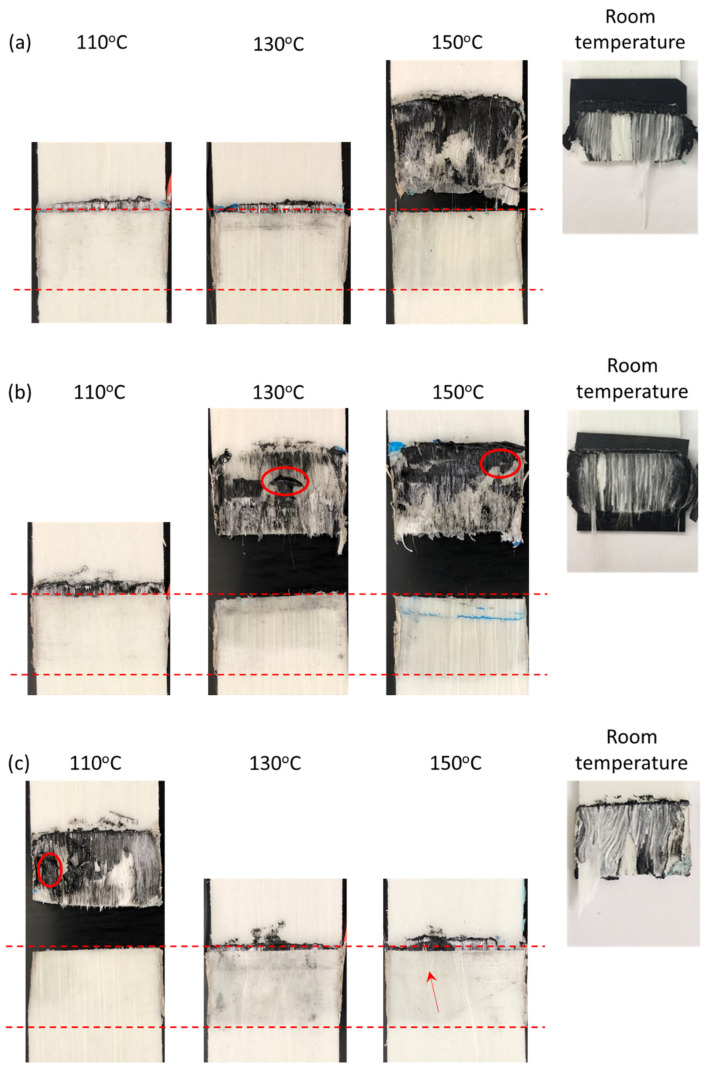
Representative photographic images of welded GF/PP adherends after disassembly procedure: (**a**) 15 wt.% MWCNT/PP film; (**b**) 20 wt.% MWCNT/PP film, and; (**c**) 25 wt.% MWCNT/PP film. The dashed red lines show the location of the overlap for the upper adherend. The circled areas indicate melted nanocomposite films. The arrow in (**c**) shows the location of a crack in the GF/PP adherend, damaged during the disassembly process. Room temperature images reproduced with permission from [36].

The fracture surface of the disassembled joints was visually observed after the procedure (Figure 11). Samples are shown as they were immediately after the tests; the welds were not manually separated to avoid influencing the appearance of the fractured joints. The red dashed lines indicate the location of the overlap under heating. Comparison with fracture surfaces at room temperature is shown on the right-hand side. Due to the temperatures reached during disassembly, all welds exhibited ply squeeze out (fiber squeeze out with polymer), as more clearly observed right above the upper dashed line in Figure 11a (110 °C and 130 °C), Figure 11b (110 °C), and Figure 11c (130 °C and 150 °C). The visible fracture surfaces exhibit a combination of cohesive and substrate failure modes: intralaminar failure through upper plies, broken fibers in the GF/PP adherend, and failure within the nanocomposite film. The MWCNT/PP films melted at the interface, with the most visible examples marked by the red circled areas in Figure 11b,c.

**Figure 12:**
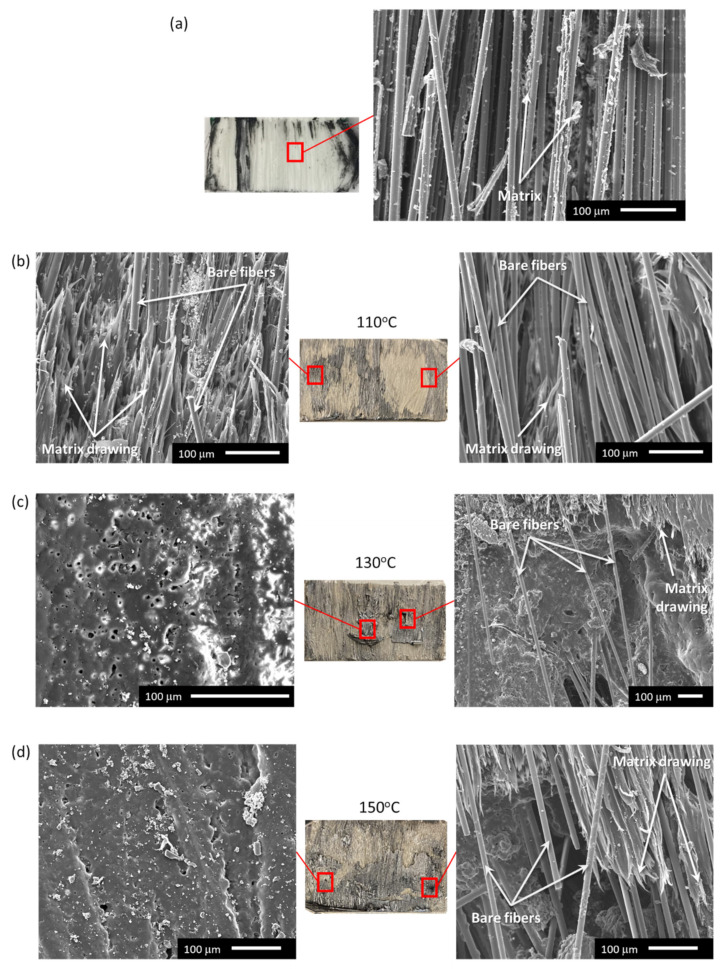
Representative fracture surfaces and SEM micrographs of samples welded with 20 wt.% MWCNT/PP films after disassembly process: (**a**) comparison with room temperature fracture surface images, reproduced and modified from [36] with permission; (**b**) disassembly at 110 °C; (**c**) disassembly at 130 °C; (**d**) disassembly at 150 °C. All scale bars are 100 μm.

Figure 12 shows SEM micrographs of fracture surfaces from Figure 11b to further analyze microstructure. Welds fractured at room temperature (Figure 12a) mostly displayed broken fibers from the upper plies of the GF/PP adherends, with matrix-fiber debonding and some matrix torn from the fibers’ surface. When disassembly temperature increased from 110 °C to 150 °C (Figure 12b–d), fracture surfaces also showed bare, broken fibers, but the thermoplastic matrix exhibited severe softening and drawing behavior with areas that underwent melting and separation from the fibers. As observed in Figure 11b,c, the presence of melted nanocomposite film was confirmed (left-hand SEM images in Figure 12c,d). The porous morphology was shown to be characteristic of the PP matrix under strain [55]. Overall, disassembly at high temperature was facilitated by a combination of melted nanocomposite film at the interface, matrix softening in the adherends, and fiber–matrix debonding.

**Figure 13:**
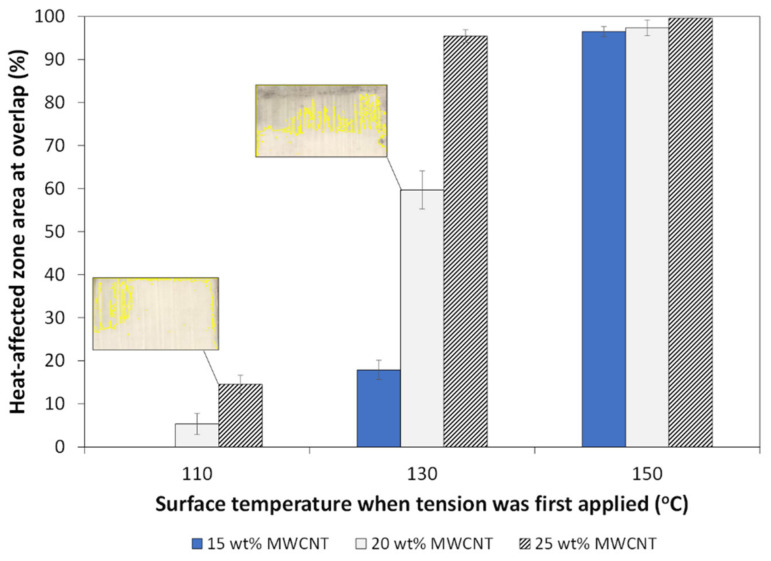
Estimated heat-affected zone area in GF/PP adherends after disassembly procedure at difference surface temperatures, based on adherends’ surface color images presented in Figure 11. Two examples of delineated areas are shown in insets.

An indication of the extent of the heat-affected zone through the GF/PP adherends’ thickness is their change in color and opacity (between the dashed, red lines). The polypropylene matrix became transparent at its melting point (150 °C), which is more clearly recognized in Figure 11b at 130 °C and 150 °C. The heat-affected area was quantified using image analysis and is reported in Figure 13 for three images under each parameter combination. The HAZ area increased by up to 97% with MWCNT content and with initial surface temperature.

### 4.3. Discussion on Disassembly Method and Its Limitations

In this study, it was observed that disassembly temperature, controlled via resistance heating, has an impact on the shear stress of ultrasonically welded joints and their HAZ. This section compares the mechanical behavior, microstructure and HAZ of specimens disassembled at every temperature. The lap shear stress was strongly influenced by the disassembly temperature with a drop up to 74% at 110 °C, 93% at 130 °C and 94% at 150 °C, compared to room temperature (Figure 9). Unlike fracture surfaces at room temperature, as shown in Figure 11, disassembled joints displayed a less uniform surface, indicating the interface reached the melting point, affecting both the nanocomposite film at the interface and the adherends’ upper plies. The MWCNT/PP films melted at the bond line, with the most visible examples marked by the red circled areas in Figure 11b,c. 

This non-uniformity was confirmed through SEM micrographs, where significant matrix softening and drawing was noted (Figure 12), exhibiting ductile failure. The melting of the nanocomposite film was mostly observed at higher temperatures (Figure 12c,d), with the presence of porosity under temperature and strain increase [55]. Similar matrix drawing was observed for carbon fiber (CF)/PPS joints tested at temperatures above T_g_, 120 °C and 150 °C [56,57]. The joints were manufactured through ultrasonic or resistance welding. In the former case, substantial matrix drawing and ductile fracture was confirmed through SEM micrographs, leading to a decrease in lap shear strength.

The HAZ reported in Figure 11, then quantified in Figure 13, is consistent with the temperature curves in Figure 10b,c, where a temperature above 150 °C was reached on the adherend’s surface during the disassembly phase. Nonetheless, the experiments confirm resistance heating can facilitate disassembly of ultrasonically welded TPC joints through a manual process, especially at higher weight fractions (20 wt.% and 25 wt.% MWCNT) and surface temperatures (130 °C and 150 °C). Under the parameters investigated in this study, welds disassembled at a surface temperature of 130 °C with 20 wt.% MWCNT present the best balance between required shear stress and heat-affected zone.

Given the results presented in this study, a discussion on the limitations of this disassembly method and future work is warranted. It was demonstrated that resistance heating through an electrically conductive nanocomposite film at the welded interface can facilitate joint disassembly by lowering the required shear stress by more than 90%. However, as the process is relatively slow (<120 s heat-up phase) and the total interface/adherends thickness is low (<4 mm), the heat-affected zone extended through the thickness, mostly at higher temperatures (130 °C and 150 °C). Consequently, disassembly was not uniquely concentrated at the bond line where the MWCNT/PP film was placed, but affected the GF/PP adherends at the overlap as well. Thus, the method might be better suited for recycling at end-of-life or reuse of components by cutting off the damaged overlap section.

Finally, as the interface was structurally compromised during disassembly, it partially affected the efficiency of resistance heating. It is expected that a faster cross-head speed during disassembly, use of highest MWCNT weight fractions (such as 20 wt.% or 25 wt.%) and control of the applied voltage during the process could mitigate this limitation, as well as the extent of the HAZ. A faster cross-head speed would reduce the time between the beginning of the disassembly phase and the peak in the stress curves (Figure 10), as well as the overall duration of the disassembly phase. Therefore, the temperature when failure initiates and propagates at maximum stress would be lower, potentially limiting the HAZ in the adherends. Further, if failure were to occur at a faster rate, the interface might not have time to cool down due to lower heating efficiency. Some future research directions include (1) investigation of disassembly parameters (e.g., crosshead speed, voltage regulation through constant power output [39]); (2) use of thicker adherends to investigate HAZ; (3) healing of bond-line defects/damage through resistance heating.

## 5. Conclusions

In this work, it was demonstrated that resistance heating via an electrically conductive MWCNT/PP film at the welded interface facilitated ultrasonic joint disassembly of TPCs. Three MWCNT fractions were characterized for thermo-electrical behavior with applied voltages up to 10V. A maximum temperature of 120 °C was reached at the highest MWCNT loading and applied voltage. For disassembly experiments, tests were initiated when the surface temperature of the GF/PP adherend reached either 110 °C, 130 °C or 150 °C. The shear stress during disassembly decreased by at least 93% at the highest MWCNT weight fraction and surface temperature, compared to room temperature testing. Analysis of fracture surfaces after disassembly revealed the melting of both MWCNT films and the adherends’ matrix at the overlap with significant matrix drawing and fiber–matrix debonding, effectively facilitating disassembly. At higher temperatures and MWCNT weight fractions, the heat-affected zone extended through the thickness of the adherends, owing to the low cross-head speed and the duration of the disassembly phase (<120 s) during which heat transfer occurred. In order to minimize the extent of the heat-affected zone area (<60%), while maximizing ease of assembly, a surface temperature of 130 °C with 20 wt.% MWCNT films would be recommended for the parameters investigated in this study.

Overall, this study confirmed the feasibility of this disassembly method for the first time in the literature. However, this might be better suited for recycling at end-of-life or reuse of components by cutting off the heat-affected overlap section. Moreover, as the interface was structurally compromised during disassembly, it affected the efficiency of resistance heating. Faster crosshead speeds during disassembly, the use of the highest MWCNT weight fractions, and control of the applied voltage during the process could, however, mitigate these limitations. 

## Figures and Tables

**Table 1:** Main estimated GF/PP adherend and MWCNT/PP films thermal properties used in FEA. Refer to Figure 4a for coordinate system.

**GF/PP**					
*V_GF_* (%)	*k_GF_* (W/m·K)	*k_PP_* (W/m·K)	*k_y_* (W/m·K)	*k_x_*, *k_z_* (W/m·K)	*C_p_* (J/kg·K)
60 ^a^	1.05 ^b^	0.15 ^a^	0.69	0.31	1.22
**MWCNT/PP**					
MWCNT wt (%)	*k_CNT_* (W/m·K)	*k_PP_* (W/m·K)	*k_CNT/PP_* (W/m·K)	*C_p_* (J/kg·K)	
15/20/25	3000 ^c^	0.15 ^a^	0.55 to 0.65 ^c^	1.50 ^c^	

a: Suppliers’ specifications sheet (PolyOne and Professional Plastics); b: [5]; c: [47,48].

## Data Availability

Not applicable.
